# Intranasal and intracerebroventricular delivery of metabolically glycoengineered neural stem cells to enhance post–cardiac arrest brain recovery

**DOI:** 10.4103/NRR.NRR-D-25-00696

**Published:** 2025-12-30

**Authors:** Xiao Liu, Zhulin Wang, Jian Du, Songah Chae, Subash Marasini, Madelynn McElroy, Kevin J. Yarema, Xiaofeng Jia

**Affiliations:** 1 Department of Neurosurgery, University of Maryland School of Medicine, Baltimore, MD, USA; 2 Department of Biomedical Engineering, The Johns Hopkins School of Medicine, Baltimore, MD, USA; 3 Translational Cell and Tissue Engineering Center, The Johns Hopkins School of Medicine, Baltimore, MD, USA; 4 Department of Orthopedics, University of Maryland School of Medicine, Baltimore, MD, USA; 5 Department of Anatomy and Neurobiology, University of Maryland School of Medicine, Baltimore, MD, USA

**Keywords:** cardiac arrest, intracerebroventricular, intranasal delivery, ischemic brain injury, metabolic glycoengineering, neural stem cell, neuroregeneration, stem cell therapy

## Abstract

Cardiac arrest leads to global cerebral ischemia, causing significant neurological deficits with few effective treatments currently available. Neural stem cell (NSC) transplantation has shown therapeutic promise; however, challenges remain to achieve optimal and consistent delivery while maximizing cell functionality post-transplantation. Metabolic glycoengineering (MGE) has emerged as a novel technique to improve NSC viability and therapeutic efficacy by modifying their glycans. This report compares the effects of different delivery routes for this innovative approach of using MGE-modified NSCs (MGE-NSCs) to treat ischemic brain injury post–cardiac arrest. A total of 16 rats were subjected to 9-minute asphyxia cardiac arrest (4 weeks) and 21 rats subjected to 11-minute asphyxia cardiac arrest (3 days) to model severe and very severe brain injury, respectively. The MGE-NSCs were administered either intranasally or intracerebroventricularly (ICV) 3 hours after resuscitation. Neurological deficits and neurobehavior tests were assessed periodically after resuscitation. Neuronal damage was examined with Fluro-Jade C staining and cresyl violet staining. The transplanted MGE-NSCs were tracked using immunofluorescence staining. In the early phase of recovery, the ICV administration resulted in better neurological deficit scores shortly after resuscitation compared with the intranasal route. Three days post-resuscitation, MGE-NSCs were primarily located in the cortex and hippocampus, with a higher percentage in the ICV-treated group. However, by 4 weeks, rats treated with intranasal MGE-NSCs exhibited superior locomotor function and reduced anxiety-/depression-related behaviors compared with those receiving ICV-NSC therapy. This was accompanied by greater survival and distribution of MGE-NSCs to the cerebellum and brainstem, along with reduced microglia recruitment. MGE-NSCs administered via both delivery methods enhanced vascular angiogenesis, neuron differentiation, and synaptic plasticity. Overall, intranasal delivery was as effective or potentially superior to ICV for long-term recovery, whereas ICV conferred short-term advantages. These results highlight the potential of MGE-NSC therapy to enhance post–cardiac arrest recovery, advocating for tailored treatment strategies based on specific recovery phase goals.

## Graphical abstract

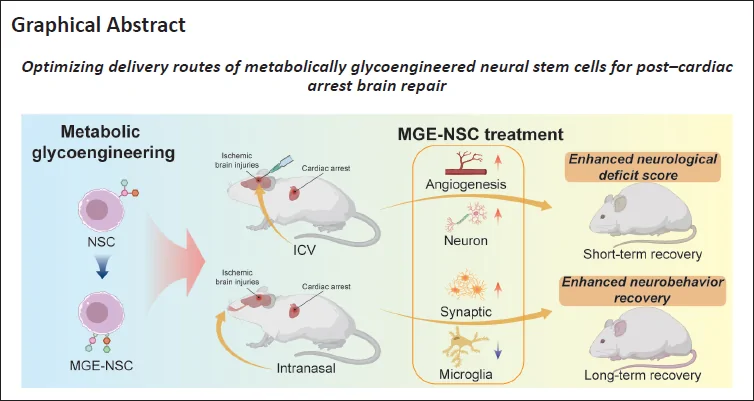

## Introduction

Cardiac arrest (CA)–induced ischemic brain injury represents a leading cause of morbidity and mortality worldwide. In-hospital CA affects an estimated 290,000 individuals and the incidence of out-of-hospital CA exceeds 350,000 annually across the United States (Holmberg et al., 2019; Virani et al., 2021); only 10%–30% of these patients survive to hospital discharge (Gräsner et al., 2020; Virani et al., 2021) with almost 50% subsequently experience enduring cognitive impairment which substantially worsens their quality of life (Caro-Codón et al., 2018; Verberne et al., 2018). CA-induced ischemic brain injury has a complex multifaceted pathology, for which effective treatments are currently unavailable. Facilitating neuro-regeneration and the restoration of neurological functions in an injured brain remains an exceedingly challenging task (Akhtar et al., 2023).

Stem cell treatment has shown promise as an improved treatment; however, its potential is significantly hindered by the restricted functionality of transplanted cells introduced to treat brain injury. Moreover, inconsistent results have been reported for various delivery methods, leaving administration routes yet to be optimized. Our team recently used metabolic glycoengineering (MGE), an approach where cellular glycans are modified by introducing non-natural monosaccharides into metabolic pathways within living cells (Du et al., 2009), to improve the therapeutic properties of neural stem cells (NSCs) (Du et al., 2021, 2022). This technique modifies the glycan composition on the surface of cells to facilitate NSC interactions with surrounding matrix materials, enhance their survival and differentiation, and improve functional outcomes after *in vivo* transplantation (Du et al., 2024).

Intracerebroventricular (ICV) administration, while invasive, directly delivers therapeutics into the cerebral spinal fluid, thereby introducing effective levels of drugs into the brain. In prior work, we showed that introducing NSCs into the lateral ventricle led to increased proliferating and migrating endogenous NSCs, protected neurons from ischemia, and promoted neurological functions in our global ischemia rodent model following cardiopulmonary resuscitation (CPR) (Wang et al., 2021). Preclinical studies of focal cerebral ischemia after stroke have also demonstrated the feasibility and effectiveness of ICV administration of stem cells (Pourmohammadi-Bejarpasi et al., 2020; Noh et al., 2021). However, the invasive nature of ICV administration can introduce additional trauma and damage to the brain parenchyma (Zhang et al., 2020; Liu and Jia, 2024a), which limits clinical translation of NSC therapies for treatment of brain injury.

Intranasal administration is an alternative treatment route for delivering therapeutics to the central nervous system. Administering substances through intranasal offers a non-invasive approach that facilitates the entry of stem cells (Wang et al., 2023) and extracellular vesicles (Lawson et al., 2022) to the brain that is practical for repeated dosages (Zhang et al., 2020; Liu and Jia, 2024a, b). The comparative efficacies of ICV and intranasal infusion of MGE-modified NSCs after CA-induced ischemic brain injury, however, have not been investigated; as a result, the therapeutic benefits or drawbacks of these two routes of administration are unknown. Accordingly, the motivation for this report is to determine the optimal delivery route for facilitating the clinical translation of MGE-NSCs in treating brain injury.

Unlike previous studies that only investigated whether naïve stem cells improved neurological function through systemic delivery methods (e.g., intravenous and intra-arterial) compared with a single direct route (ICV) (Zhou et al., 2014; Leong et al., 2016), the current study explored two direct delivery routes to the brain (i.e., intranasal *versus* ICV administration). Furthermore, the current study extended exploration of administration options beyond naïve NSCs to MGE-modified NSCs, elucidated the fate of MGE-NSCs after transplantation, and finally, compared for the first time a severe (9-minute resuscitation interval) with a very severe (11-minute resuscitation interval) brain injury model. To demonstrate efficacy, we assessed various functional and behavioral parameters, comparing the therapeutic advantages of MGE-NSCs between different delivery routes post-CA. Finally, we observed no tumorigenesis or severe adverse events associated with engrafted MGE-NSCs, thus alleviating safety concerns. Overall, in both severe and very severe ischemic brain injury models induced by CA, this study highlights a differential effect of MGE-NSC delivery routes, with ICV administration proving more beneficial in the short term and intranasal delivery offering greater advantages over the long term. These results establish a promising and easily accessible biotherapy in clinical neuroscience for mitigating the short- and long-term effects of CA-induced ischemic brain injury.

## Methods

### Neural stem cell culture and metabolic glycoengineering modification

Tissue culture plates were prepared for NSC (ReNcellVM Human Neural Progenitor Cell Line) culture by coating with Poly-L-Lysine (10 µg/mL, Sigma, St. Louis, MO, USA) at 37°C for 2.0 hours followed by coating with laminin (5 µg/mL, Sigma) overnight at room temperature. Culturing continued with basic fibroblast growth factor (20 ng/mL, Thermo Fisher Scientific, Waltham, MA, USA) and epidermal growth factor (20 ng/mL, Thermo Fisher Scientific) in the Maintenance Medium (Millipore, Burlington, MA, USA). Cell seeding commenced on day 1, followed by the addition of the custom-synthesized MGE sugar analog Ac_5_ManNTProp to the NSC medium on day 2, with subsequent media replenishment every other day (Du et al., 2024); Ac_5_ManNTProp was synthesized, purified, and characterized using methods detailed previously (Du et al., 2021).

### Experimental animals

The Institutional Animal Care and Use Committee of the University of Maryland, Baltimore approved all animal experimentation procedures. Adult Wistar rats (age: 10–12 weeks; body weight: 270–370 g) were used in this study. Rats were housed in rat conventional cages (2–3 animals per cage before surgery) under controlled environmental conditions (temperature (22 ± 2°C, relative humidity 30%–70% and a 12-hour light/dark cycle), with free access to standard chow and water. Using randomization, twenty-one adult Wistar rats (Charles River, Wilmington, MA, USA) that underwent 11-minute asphyxia CA were allocated into three experimental conditions for short-term study (3 days) (3 male/4 female, *n* = 7): the first group was treated with MGE-NSCs intranasally (intranasal group), a second group was treated with MGE-NSCs intracerebroventricularly (ICV group), and the CA only (CTL group) received phosphate-buffered saline (PBS) without transplanted NSCs. We also employed a random assignment procedure to divide sixteen adult Wistar rats (Charles River) into two groups for long-term study (4 weeks) (8 male, *n* = 8): rats were subjected to 9-minute asphyxia CA before receiving treatment with MGE-NSCs intranasally (intranasal group), rats were subjected to 9-minute asphyxia CA before receiving treatment with MGE-NSCs intracerebroventricularly (ICV group). Rats were excluded if, after 2 minutes of CPR, the return of spontaneous circulation (ROSC) was not attained. Because NSCs were administered 3 hours after ROSC, rats which died within 3 hours post-ROSC were also excluded.

### Asphyxia cardiac arrest model

**Figure 1:**
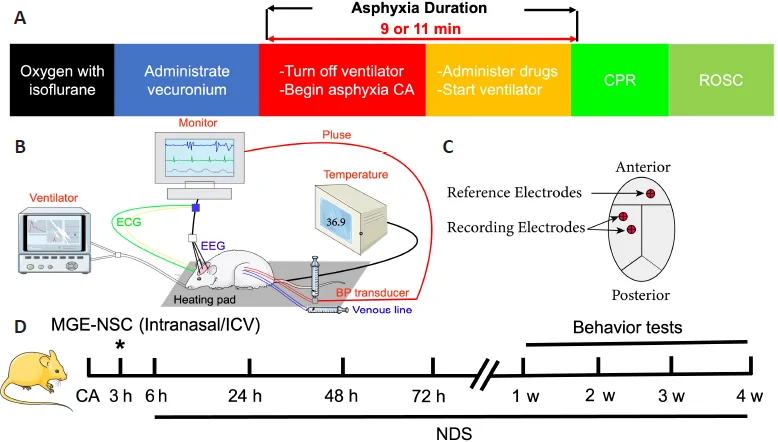
Schematic of experimental design. (A) Establishment of 9-minute (min) asphyxia CA-induced severe brain injury and 11-min asphyxia CA-induced very severe brain injury. (B) Continuous monitoring and recording of EEG, ECG, blood pressure and body temperature throughout the experiment. (C) Electrodes were implanted for EEG recording. (D) MGE-NSCs were transplanted 3 hours post-ROSC. NDS and behavior tests were performed at multiple time points. BP: Blood pressure; CA: cardiac arrest; CPR: cardiopulmonary resuscitation; ECG: electrocardiogram; EEG: electroencephalogram; h: hour; ICV: intracerebroventricular; MGE-NSC: metabolic glycoengineering modified neural stem cell; NDS: neurological deficit scores; ROSC: return of spontaneous circulation; w: week.

The asphyxia CA model has been well established in our previous studies (Jia et al., 2006, 2008). Briefly, rats were anesthetized with 1.5% isoflurane (Covetrus, Portland, ME, USA) in 50% O_2_/50% N_2_, followed by tracheal intubation and ventilator support (RWD Life Science Co., Ltd.). Blood pressure recording and blood sampling for arterial blood gas (ABG) tests were achieved via cannulation of the right femoral artery. A similar cannulation procedure was performed in the right femoral vein for drug delivery. To induce the asphyxia, muscles were blocked by vecuronium (2 mL/kg) and tracheal tube clamping were maintained for 11 minutes in short-term study to induce survival but with very severe brain injury, and for 9 minutes in long-term study to induce severe brain injury, respectively. A mean arterial pressure (MAP) below 10 mmHg was used as the threshold for identifying CA. Resuscitation drugs (sodium bicarbonate 1 mL/kg, epinephrine 0.5 mL/kg) and continuous CPR were initiated until ROSC was achieved (defined as MAP 60 mmHg). The body temperature of all rats was maintained by a heating pad at 37 ± 0.5°C during the experiments (**Figure 1A** and **B**).

### Metabolic glycoengineering–modified neural stem cell administration

MGE-NSC administration was conducted 3 hours post ROSC intranasally (Wang et al., 2023) or intracerebroventricularly (Wang et al., 2021). In the Intranasal group, stem cells were administered in right and left nostril twice by a pipette tip to enhance the permeability. A cell suspension (10 µL) was injected into each nostril at 5-minute intervals, with a total volume of 40 µL (3 × 10^5^ NSCs) delivered intranasally. In the ICV group, rats were positioned on the stereotaxic instrument (ML: 1.4 mm, AP: –0.8 mm, DV: 3.7 mm) (Wang et al., 2021) and 3 × 10^5^ NSCs in 10 µL volume were ICV-delivered at a slow rate over a period of 5 minutes, followed by a 5-minute dwell time with the needle in place to prevent potential leakage. Rats were anesthetized during the transplantation if they had recovered from coma 3 hours after ROSC. For surgical preparation and experimental procedures, rats were anesthetized with 1.5%–2% isoflurane (Covetrus) in oxygen-enriched air, delivered via a nose cone. Adequate depth of anesthesia was confirmed by the absence of pedal withdrawal reflex before initiating the procedure.

### Electrophysiological monitoring and assessment

Electroencephalogram (EEG) monitoring has been well validated to be a useful electrophysiological method of predicting neurological outcomes after CA in our prior studies (Jia et al., 2006, 2008a, b). EEG signal was continuously recorded for 6 hours via a two-channel EEG system (Tucker-Davis Technologies, Alachua, FL, USA). The first 5-minute of EEG served as the baseline, after which CA was monitored until 6 hours post-ROSC. The EEG data were assessed in terms of quantitative EEG-information quantity (EEG-IQ) (Jia et al., 2006, 2008a, b) at baseline, CA time and 1 to 6 hours post-ROSC. All IQ data were normalized to baseline (**Figure 1B** and **C**).

### Arterial blood gas analysis

Measurements were obtained via the i-STAT1 system (Abaxis, Union City, CA, USA), femoral arterial blood samples were tested for ABG before CA and 20-minute after ROSC to analyze the respiratory and metabolic disorders (Wang et al., 2021, 2023). Following the analysis of ABGs, ventilator settings were modified to optimize respiratory status.

### Neurological function evaluation

Neurological deficit scores (NDS) were evaluated at 6, 24, 48, 72 hours, and then again 1, 2, 3, 4 weeks following ROSC (**Figure 1D**) with a range of 0–80 scores, which consisted of general behavior, brainstem function, motor assessment, sensory assessment, behavior and the presence of seizure (Jia et al., 2008a, b; Wang et al., 2021, 2023). Aggregate NDS and sub-NDS (including motor, sensory, and brainstem functions) were compared between groups. The survival time and survival rate were also analyzed to assess the neurological outcomes.

### Neurobehavior tests

The assessment of behavior function was assessed within a controlled environment featuring sound attenuation and dim lighting. Rats were evaluated at 1, 2, 3, and 4 weeks post-ROSC (**Figure 1D**). The elevated plus maze and tail suspension test were performed to evaluate the anxiety-like and depression-like behaviors as we previous published (Du et al., 2024). Both for a 5-minute session, the rearing events (rats stand on their hind legs), grooming episodes (cleaning movements on the face by forelegs), the duration remaining in the open arms and open-arm entries were analyzed in the elevated plus maze test. The total immobility time, a measure of depression-like behavior, was assessed using the tail suspension test. In addition, the catwalk evaluation, which offers a sensitive approach to assess locomotor function, was performed to evaluate locomotor functional recovery following our published protocol (Du et al., 2018; Zhang et al., 2021a). By using the gait analysis system (CatWalk XT; Noldus), all four paws were recognized and recorded automatically. Each animal completed a minimum of three successful runs. The speed and maximum intensity were calculated.

### Tissue preparation and sectioning

At the designated time points, animals were deeply anesthetized with 30% urethane until a stable, deep plane of anesthesia was confirmed by loss of pedal and corneal reflexes. Animals were then transcardially perfused first with ice-cold PBS to clear blood, followed by 4% paraformaldehyde in PBS as a fixative. Brains were removed and post-fixed in 4% paraformaldehyde at 4°C for 24 hours, then transferred to 30% sucrose in PBS at 4°C until the tissue sank (cryoprotection). For frozen sections, brains were embedded in OCT compound and rapidly frozen on dry ice. Coronal sections (15 µm thickness) were cut on a cryostat at –20°C and collected on glass slides.

### Cresyl violet staining

Neuron ischemia in the regions of dentate gyrus (DG), CA3, and CA1 were evaluated with cresyl violet staining (Wang et al., 2023). All coronal slides of the hippocampus were evaluated under Leica DMi8 microscope (Leica Microsystems, Wetzlar, Germany). Dead/survival neurons in DG, CA3, and CA1 were quantified.

### Fluoro-Jade C staining

Degenerating neurons were identified using Fluoro-Jade C (FJC) staining, following the protocol provided with the Fluoro Jade C Ready to Dilute Staining Kit (Cat# TR-100-FJT, Biosensis, Temecula, CA, USA) (Ikenari et al., 2020). In summary, brain slides were prepared using 80% ethanol, sodium hydroxide, and potassium permanganate, following instructions supplied with the kit. They were subsequently stained for 10 minutes in the dark using a mix of FJC and 4′,6-diamidino-2-phenylindole (DAPI) solutions (Millipore), diluted 1:10 from stock solutions. Fluorescent images were captured using a fluorescence microscope (Leica microsystem), and the overall area and intensity of FJC-positive cells were quantified using ImageJ software (ImageJ 1.53k).

### Immunofluorescence staining

Immunofluorescence staining was performed with 15-μm thick frozen brain sections. For cell tracking, mouse anti-human mitochondria (1:200, Millipore, MAB1273) and STEM101 (human nucleus, 1:100, Takara Bio, San Jose, CA, USA) antibodies were selected to monitor transplanted MGE-NSC distribution. To examine MGE-NSC engulfment by microglia, primary antibody rabbit anti-ionized calcium-binding adapter molecule 1 (Iba1, a microglia marker, 1:500, Abcam, Cambridge, UK, Cat# AB178846, RRID: AB_2636859) was introduced with mouse anti-human mitochondria (1:200, Millipore, Cat# MAB1273, RRID: AB_94052). We employed immunostaining for rabbit anti-MAP2 (a neuron marker, 1:500, Sigma-Aldrich, Cat# M3696, RRID: AB_1840999) with mouse anti-human mitochondria (1:200, Millipore, Cat# MAB1273, RRID: AB_94052) to investigate cell differentiation in transplanted cells. Mouse anti-RECA1 (a vascular endothelium marker, 1:200, Novus Biologicals, Cat# NB100-64647, CO, USA) was used with rabbit anti-Ki67 (a proliferation marker, 1:200, Abcam, Cat# ab16667, RRID: AB_302459) to measure the angiogenesis and function of vascular structure. Rabbit anti-synapsin 1 (a mature presynaptic marker, 1:500, Millipore, Cat# AB1543, RRID: AB_2200400) was used to assess synaptic plasticity. Immunofluorescence imaging were performed on a Leica DMi8 microscope (Leica Microsystems). Quantifications were conducted using ImageJ.

### Statistical analysis

All statistical computations were performed using IBM SPSS Statistics (version 26). Nonparametric testing (Kruskal-Wallis one-way analysis of variance [k samples]) was performed to analyze NDS, which were expressed as median and 25^th^–75^th^ interquartile range. Statistical analysis of parametric values included a one-way analysis of variance and subsequent *post hoc* testing (Tukey or Least Significant Difference), with results expressed as mean ± SEM. The threshold for statistical significance was established at *P* < 0.05.

## Results

### Baseline characteristics

**Table 1** shows the characteristics of baseline ABG before CA. There were no significant differences between the intranasal and ICV groups in terms of partial pressures of gas in blood (partial pressure of carbon dioxide [PCO_2_], partial pressure of oxygen [PO_2_], oxygen saturation [SO_2_], total carbon dioxide [TCO_2_]) and acid-base content (bicarbonate [HCO_3_^–^], lactate). All the baseline conditions were similar between different groups (*P* > 0.05).

**Table 1:** Baseline arterial blood gas in the intranasal and ICV groups for long-term study

	Intranasal	ICV	*P*-value
pH	7.34 ± 0.12	7.34 ± 0.11	0.706
PCO_2_ (mmHg)	44.09 ± 2.74	37.63 ± 2.55	0.707
PO_2_ (mmHg)	242.83 ± 8.66	242.50 ± 9.30	0.888
Base excess	–1.87 ± 0.85	–3.87 ± 1.12	0.379
HCO_3_^–^ (mM)	23.81 ± 0.95	20.09 ± 1.23	0.466
TCO_2_ (mmHg)	25.25 ± 1.01	21.25 ± 1.38	0.402
SO_2_ (%)	100 ± 0.00	100 ± 0.00	–
Lactate (mM)	0.93 ± 0.08	0.66 ± 0.03	0.061

Data are expressed as mean ± SEM. ICV: Intracerebroventricular.

### Electroencephalogram-information quantity recovery

During CA time, the regular EEG signal transitioned into an isoelectric trace, marked by a sharp decrease in IQ value to almost zero. Following resuscitation, both EEG amplitude and EEG-IQ values gradually recovered. The responses of intranasal and ICV groups remained statistically similar across all temporal observations (*P* > 0.05; **Additional Figure 1A** and **B**). Representative EEG waveforms were displayed in **Additional Figure 1C**.

### Neurological function recovery

**Figure 2:**
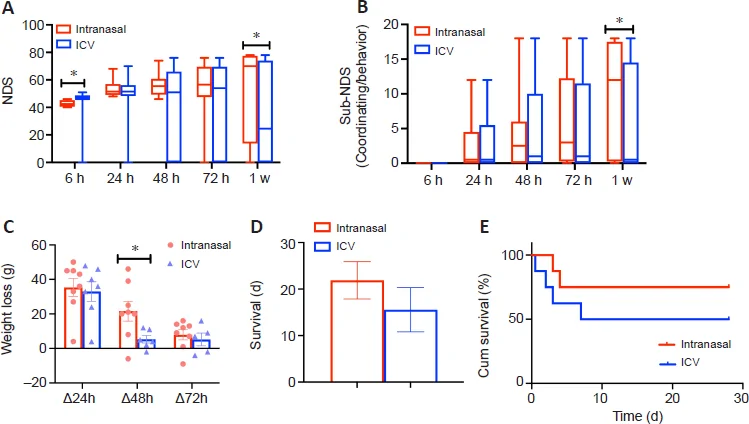
MGE-NSC therapy shows the advantages of short-term and long term-effects through ICV and intranasal routes, respectively. (A, B) Assessment of aggregated NDS (A) and sub-NDS of motor function (B) after cardiac arrest–induced severe brain injury between intranasal and ICV groups. (C) Weight loss (g) during every 24-hour at first 3 days after cardiopulmonary resuscitation/cardiac arrest. Weight loss Δ24h = body weight_24h_ – body weight_Baseline_; weight loss Δ48h = body weight_48h_ – body weight_24h_; Weight loss Δ72h = body weight_72h_ – body weight_48h_. (D, E) Survival time (D) and 28-day cumulative survival analysis (E) of intranasal-NSCs and ICV-NSCs treated rats. Data in A and B are depicted as median, 25^th^–75^th^ percentile (*n* = 8 animals/group), *P < 0.05 (nonparametric test of Kruskal-Wallis one-way ANOVA). Data in C and D are presented as mean ± SEM (*n* = 8 animals/group), *P < 0.05 (one-way analysis of variance). Data in E are presented as cumulative (cum) survival rate (*n* = 8 animals/group), using Kaplan-Meier method. h: Hour; ICV: intracerebroventricular; MGE-NSC: metabolic glycoengineering modified neural stem cell; NDS: neurological deficit scores; w: week.

In the short-term study 3 days after 11-minute CA, both the intranasal and ICV treatment groups had higher NDS than the control group. In the long-term study 4 weeks after 9-minute CA, the ICV group demonstrated superior early recovery based on 6-hour NDS results (intranasal *versus* ICV: *P* = 0.045; **Figure 2A** and **Additional Table 1**). Conversely, the intranasal group presented higher NDS recovery at a later time point; specifically, 1 week after ROSC (intranasal *versus* ICV: *P* = 0.038; **Figure 2A** and **Additional Table 1**), accompanied by significantly better advanced motor recovery (intranasal *versus* ICV: *P* = 0.042; **Figure 2B** and **Additional Table 2**). Notably, the two groups exhibited similar patterns regarding sensory and brainstem function sub-NDS (**Additional Figure 2** and **Additional Tables 3**, **4**). The 72-hour survival rate showed no statistical differences among intranasal (5/7, 71%), ICV (4/7, 57%) and the CTL groups (4/7, 57%; **Additional Figure 3**).

**Additional Table 1:** Neurological deficit scores in the intranasal and ICV groups

	Intranasal	ICV	*P* value
6 h	42.50 (40.50-45.25)	46.00 (46.00-48.75)	0.045*
24 h	51.50 (49.00-57.00)	51.50 (48.25-56.25)	0.798
48 h	55.50 (49.25-60.75)	51.00 (0.00-66.00)	0.574
72 h	56.50 (47.50-69.50)	54.00 (0.00-69.50)	0.645
1 wk	70.00(13.75-77.50)	21.50 (0.00-74.00)	0.038*

Data are expressed as median (25^th^-75^th^ interquartile range). *P < 0.05; *n* = 8 at each time point. ICV: Intracerebroventricular.

**Additional Table 2:** Sub-neurological deficit scores (motor) in the intranasal and ICV groups

	Intranasal	ICV	*P* value
6 h	0.0 (0.0-0.0)	0.0 (0.0-0.0)	1.000
24 h	0.5 (0.0-4.5)	0.5 (0.0-5.5)	0.955
48 h	2.5 (0.0-6.0)	1.0 (0.0-10)	0.912
72 h	3 (0.25-12.25)	1.0 (0.0-11.5)	0.589
1 wk	8 (0.25-17.5)	0.5 (0.0-14.5)	0.042*

Data are expressed as median (25^th^-75^th^ interquartile range). *P < 0.05; *n* = 8 at each time point. ICV: Intracerebroventricular.

**Additional Table 3:** Sub-neurological deficit scores (sensory) in the intranasal and ICV groups

	Intranasal	ICV	*P* value
6 h	2.0 (1.25-2.0)	2.0 (1.25-2.0)	0.878
24 h	4.0 (2.0-4.0)	3.0 (2.0-4.0)	0.798
48 h	4.0 (4.0-4.0)	3.0 (0.25-4.0)	0.328
72 h	4.0 (4.0-5.5)	3.0 (0.0-6.0)	0.645
1 wk	6.0 (1.0-6.0)	1.5 (0.0-6.0)	0.328

Data are expressed as median (25^th^-75^th^ interquartile range). *n* = 8 at each time point. ICV: Intracerebroventricular.

**Additional Table 4:** Sub-neurological deficit scores (brainstem function) in the intranasal and ICV groups

	Intranasal	ICV	*P* value
6 h	12.0 (9.75-14.25)	15.0 (12.75-18.0)	0.083
24 h	19.5 (15.75-21.0)	18.0 (18.0-18.0)	0.442
48 h	21.0 (15.75-21.0)	18.0 (0.0-21.0)	0.279
72 h	21.0 (14.25-21.0)	21.0 (0.0-21.0)	0.574
1 wk	21.0 (5.25-21.0)	7.5 (0.0-21.0)	0.279

Data are expressed as median (25^th^-75^th^ interquartile range). *n* = 8 at each time point. ICV: Intracerebroventricular.

Comparison of body weights revealed that the ICV group experienced more pronounced weight loss during the second 24-hour period after ROSC (intranasal *versus* ICV: *P* = 0.038; **Figure 2C** and **Additional Table 4**); subsequently, gradual recovery occurred with the body weights of the groups becoming similar over time. The evaluation survival times and 28-day survival rates suggested more favorable results in subjects receiving intranasal treatment (6/8, 75%) compared with those in the ICV group (4/8, 50%). However, this observed difference was not statistically significant (**Figure 2D** and **E**).

No tumorigenesis or severe adverse reactions related to cell transplantation were observed during the 4-week follow-up period. Complications commonly accompanying CA with severe brain injury, such as fever and seizure, were observed at statistically similar levels across both groups: 2 out of 8 rats in the intranasal group and 3 out of 8 in the ICV group experienced high fever (rectal temperature > 39°C) and seizure. The seizure symptoms resolved upon fever control with acetaminophen and no additional antiepileptic drugs were administered.

### Neurobehavior results

**Figure 3:**
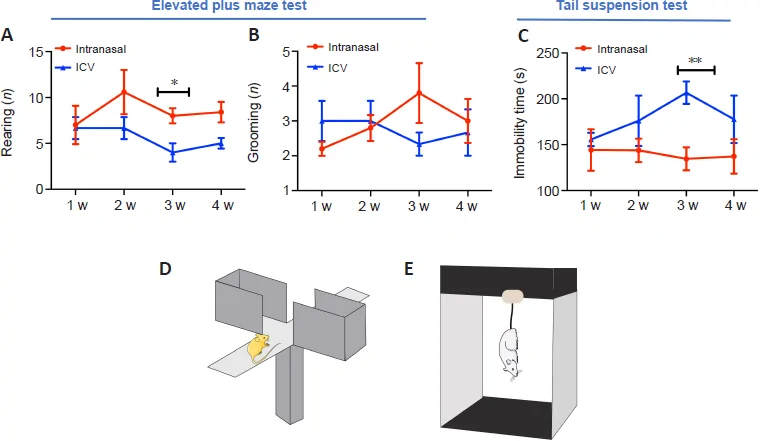
Chronic behavior tests after severe brain injury induced by cardiac arrest. (A, B) Anxiety-associated behaviors of rearing (A) and grooming events (B) assessed in the elevated plus maze test. (C) Depression-associated behaviors of immobility in the tail suspension test. (D, E) The diagram of anxiety-related behavior in the elevated plus maze (D) and suppression-related behaviors in the tail suspension test (E). Data were presented as mean ± SEM (*n* = 4–5 animals/group). *P < 0.05, **P < 0.01 (one-way analysis of variance). ICV: Intracerebroventricular; w: week(s).

We performed the elevated plus maze test, as described in our studies (Du et al., 2024), to evaluate anxiety-related behaviors. Three weeks after cell transplantation, the ICV group exhibited fewer rearing events, indicative of standing on hind legs, compared with the intranasal group (intranasal *versus* ICV: *P* = 0.024; **Figure 3A**), suggesting a higher propensity for anxiety in the ICV group. However, both the ICV and intranasal groups showed similar frequency of grooming events, including paw washing, face washing, and body washing by the rats (*P* > 0.05; **Figure 3B**).

Additionally, the tail suspension test was conducted to measure depression-like behaviors (Du et al., 2024). The results demonstrated that the duration time of immobility was noticeably prolonged in the ICV group compared with the Intranasal group at 3 weeks post-ROSC (intranasal *versus* ICV: *P* = 0.009; **Figure 3C**), indicating a higher occurrence of depression-associated behaviors in the ICV group.

**Figure 4:**
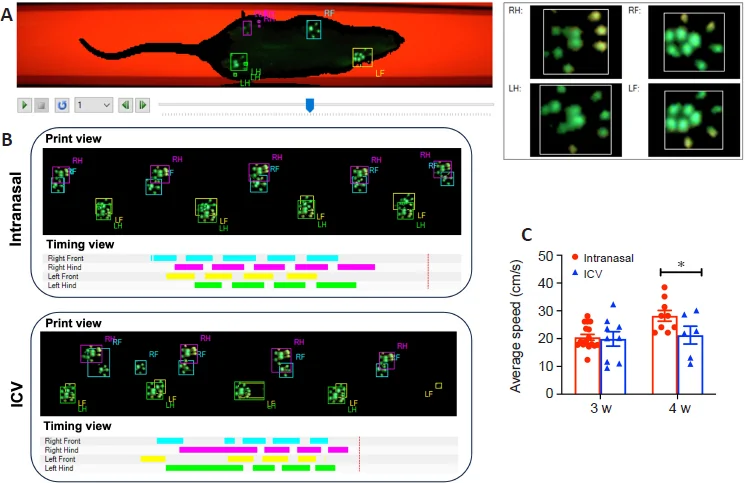
Locomotor functional recovery in Catwalk evaluation after cell transplantation. (A) Representative figure of the CatWalk XT gait analysis system. The footprints of four paws were recorded and average speed and total time of running were analyzed automatically. (B) Representative figure of print view and timing view in the intranasal and ICV groups. Prolonged timing was observed in ICV group compared with the intranasal group (red and blue bold lines). (C) Analysis of average speed between the intranasal and ICV groups at different time points after cardiopulmonary resuscitation/cardiac arrest. Data were presented as mean ± SEM (*n* = 4–5 animals/group). *P < 0.05 (one-way analysis of variance). ICV: Intracerebroventricular; LF: left front; LH: left hind; RF: right front; RH: right hind.

Furthermore, the Catwalk gait analysis assay (**Figure 4A** and **B**) showed rats in the intranasal group had faster movement speeds at 4 weeks post-CA compared with those in the ICV group (intranasal *versus* ICV: *P* = 0.03; **Figure 4C**).

### Metabolic glycoengineering-modified neural stem cells administration promotes the survival of neuronal cells in the hippocampus

**Figure 5:**
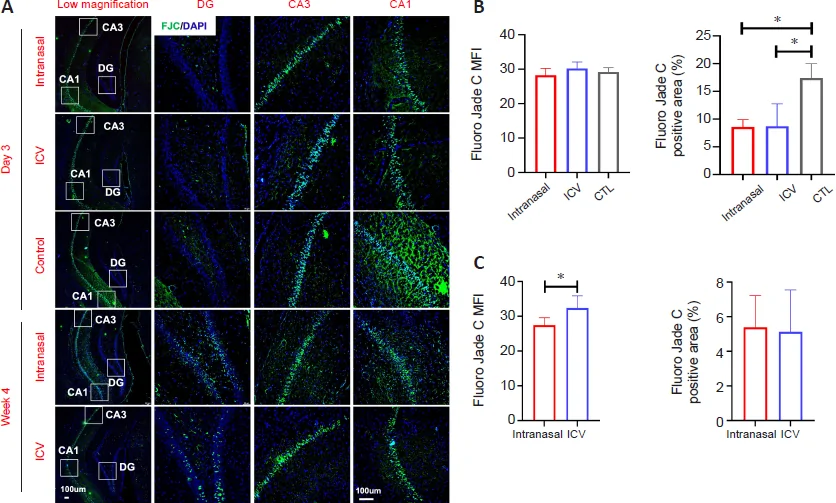
Neuronal degeneration in the hippocampus of rats at 3 days and 4 weeks post-transplantation. (A) Representative images of Fluoro-Jade C (FJC, green) and DAPI (blue) staining in the hippocampal regions (DG, CA1, and CA3) from different groups at 3 days and 4 weeks post-cardiac arrest. Images at low magnification show the different hippocampal regions (DG, CA1, and CA3). FJC-positive cells indicate degenerating neurons, and DAPI labels all cell nuclei. (B, C) Quantitative analysis of FJC staining at 3 days and 4 weeks post–cardiac arrest. Bar graphs show the mean fluorescence intensity (MFI) and percentage of FJC-positive area in the hippocampus. Quantitative data are presented as mean ± SEM (*n* = 3 animals per group). *P < 0.05 (one-way analysis of variance). CTL: Control; DAPI: 4′,6-diamidino-2-phenylindole; DG: dentate gyrus; ICV: intracerebroventricular.

To evaluate neuronal degeneration, FJC staining was performed on brain sections from rats subjected to CA. At 3 days post-CA, the FJC-positive area percentage was substantially lower in both the intranasal NSC treatment group and the ICV NSC injection group compared with the control group (intranasal versus CTL: *P* = 0.029; ICV *versus* CTL: *P* = 0.042; **Figure 5A** and **B**), suggesting early neuroprotective effects of MGE’d NSC therapy. However, there was no significant difference in overall FJC fluorescence intensity among the groups (intranasal, ICV *versus* CTL: *P* > 0.05; **Figure 5A** and **B**). At 4 weeks post-CA, the intranasal group exhibited a significantly lower FJC fluorescence intensity compared with the ICV group (*P* = 0.013; **Figure 5A** and **C**), indicating a sustained reduction in the severity of neurodegeneration in the intranasally treated animals during the subacute recovery phase.

**Figure 6:**
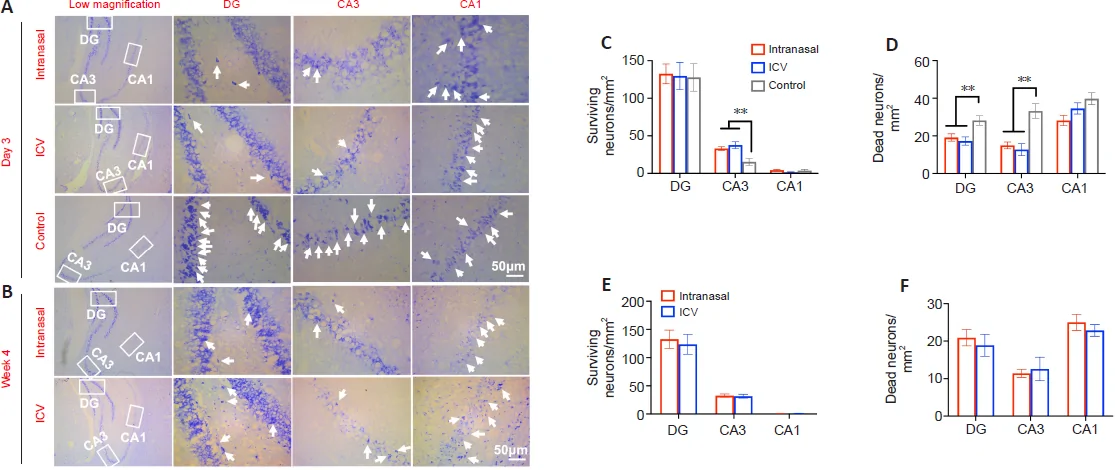
Cresyl violet staining to evaluate the ischemic neurons in the hippocampus. Survival neurons and ischemic neurons were assessed in DG, CA3, and CA1 regions. (A) Representative photographs of neurons in the hippocampus 3 days after 11-minute axphyxia cardiac arrest among the control, intranasal, and ICV groups. (B) Representative photographs of neurons in the hippocampus 4 weeks after 9-minute axphyxia cardiac arrest between the intranasal and ICV groups. White arrows indicate ischemic or dead neurons. Quantifications of the surviving neurons (C) and ischemic neurons (D) 3 days after treatment. Quantitative analysis of surviving neurons (E) and dead neurons (F) did not show significant differences between the intranasal and ICV groups at 4 weeks. Data are presented as mean ± SEM (*n* = 4–5 animals/group), **P < 0.01 (one-way analysis of variance). DG: Dentate gyrus; ICV: intracerebroventricular.

Next, to determine the ability of MGE-NSCs to provide neuronal protection, we probed the number of living neurons and dead neurons in hippocampus via cresyl violet staining. Specifically, MGE-NSC treatment in both the intranasal and ICV groups exerted neuroprotective effects in the hippocampus. Three days after cell transplantation, treatment groups showed superior survival neuron viability in CA3 compared with controls (intranasal *versus* CTL: *P* = 0.001; ICV *versus* CTL: *P* = 0.001; **Figure 6A** and **C**). Rats in MGE-NSC treatment groups also had fewer dead neurons in regions of DG (intranasal *versus* CTL: *P* = 0.006; ICV *versus* CTL: *P* = 0.004) and CA3 (intranasal *versus* CTL: *P* < 0.001; ICV *versus* CTL: *P* < 0.001; **Figure 6A** and **D**) than the CTL group. No statistical differences were detected between intranasal and ICV groups at either the 3-day (**Figure 6A**, **C**, and **D**) or 4-week post-CA (**Figure 6B**, **E**, and **F**).

### Distribution, migration, and differentiation of transplanted metabolic glycoengineering–modified neural stem cells

**Figure 7:**
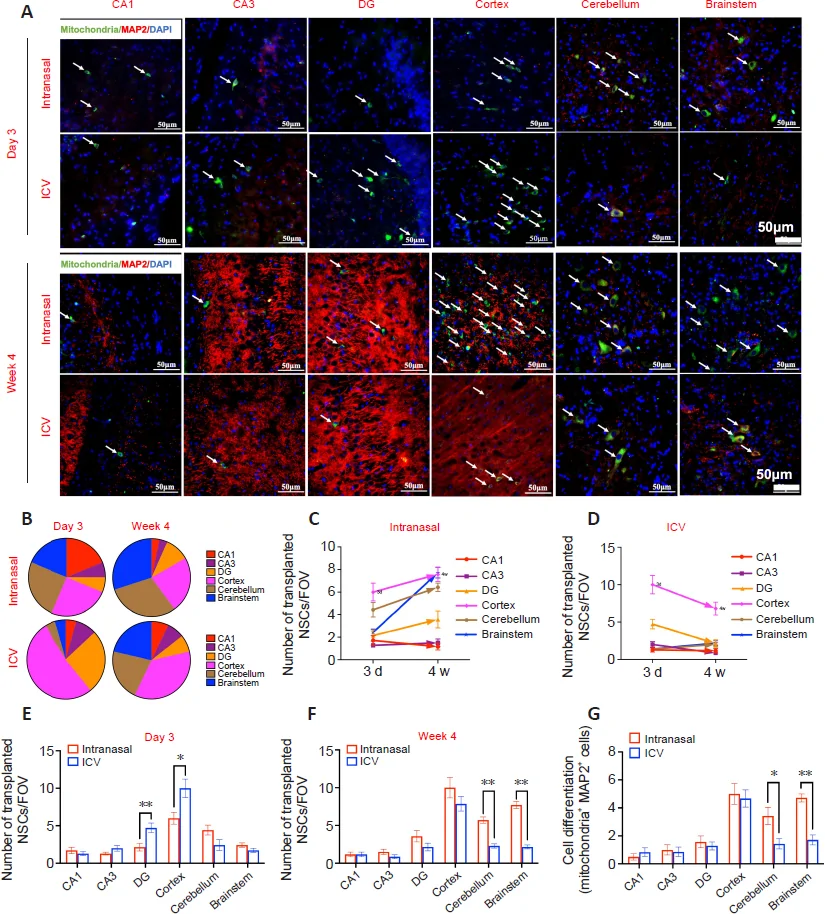
Transplanted stem cell tracking and differentiation. (A) Representative figures of human mitochondria (green) and MAP2 (red) double immunofluorescence in the intranasal and ICV groups 3 days and 4 weeks post-return of spontaneous circulation. Nuclei were counterstained with DAPI (blue). (B) The proportion of transplanted MGE-NSCs distributed in different brain regions from 3 days to 4 weeks. The trend of NSCs migrations in different regions from 3 days to 4 weeks between the intranasal (C) and ICV groups (D). (E, F) Quantification of transplanted MGE-NSCs detected in each field of view. (G) Quantitative analysis of MAP2 and human mitochondria double positive cells, indicating the transplanted MGE-NSCs differentiating into neurons. Data are presented as mean ± SEM (*n* = 4–5 animals/group). *P < 0.05, **P < 0.01 (one-way analysis of variance). DAPI: 4′,6-Diamidino-2-phenylindole; DG: dentate gyrus; FOV: field of view; h: hour; ICV: intracerebroventricular; MAP2: microtubule associated protein 2; MGE-NSCs: metabolic glycoengineering-modified neural stem cells; NSCs: neural stem cell; w: week.

In the short-term experiments involving severe brain injury induced by 9-minute CA, MGE-NSCs were found to have migrated to the hippocampus, cortex, cerebellum, and brainstem in roughly equal proportions by day 3 following intranasal transplantation. Conversely, in the ICV group, the distribution of MGE-NSCs were mainly concentrated in the hippocampal and cortical regions, with only a few cells were detected in the cerebellum and brainstem (**Figure 7A** and **B**). ICV treatment resulted in enhanced MGE-NSC accumulation within DG (intranasal *versus* ICV: *P* = 0.009; **Figure 7A**, **D**, and **E**) and cortex (intranasal *versus* ICV: *P* = 0.016; **Figure 7A**, **D**, and **E**) compared with the intranasal group.

In the long-term study involving very severe brain injury induced by 11-minute CA, fewer MGE-NSCs were observed in the ICV group on day 28 following transplantation, cellular mapping demonstrated preferential accumulation in cortical zones near the subventricular zone. In contrast, NSCs in the intranasal group migrated to more distant regions, including the cerebellum and brainstem (**Figure 7A**, **C**, and **F**). A greater number of transplanted MGE-NSCs were observed in regions of the cerebellum (intranasal *versus* ICV: *P* < 0.000; **Figure 7A**, **C**, and **F**) and the brainstem (intranasal *versus* ICV: *P* < 0.000; **Figure 7A**, **C**, and **F**) in the intranasal group compared with the ICV group.

To determine the extent of cell differentiation in MGE-NSCs administered by the two delivery methods, we examined the expression of MAP2^+^/human mitochondria^+^ cells via immunostaining. Co-immunofluorescence staining for human mitochondria and MAP2 showed a greater number of differentiated cells in the intranasal group in the cerebellum (intranasal *versus* ICV: *P* = 0.016; **Figure 7G**) and brainstem (intranasal *versus* ICV: *P* < 0.001; **Figure 7G**), indicating greater cell differentiation into neurons in the intranasal group than ICV group 4 weeks post-ROSC.

### Microglia engulfment

**Figure 8:**
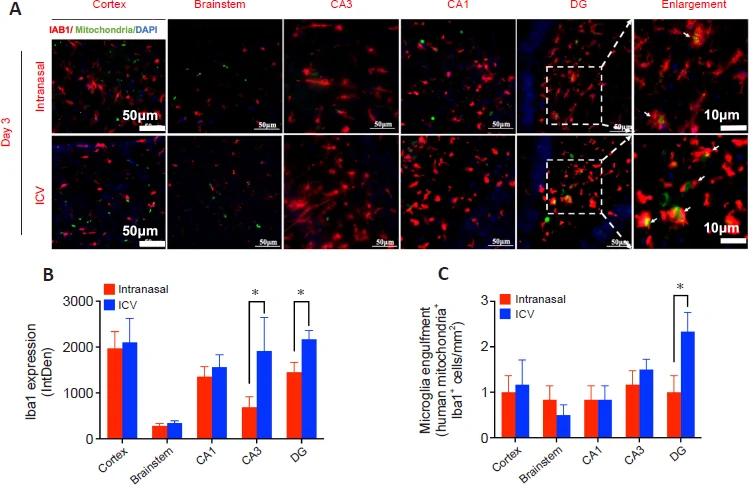
Microglia engulfment after cell transplantation. (A) Representative figures of microglia (Iba1, red) and transplanted stem cells (human mitochondria, green) in different regions 3 days after cardiac arrest/cardiopulmonary resuscitation between the intranasal and ICV groups. Nuclei were counterstained with DAPI (blue). In the enlargement figure, activated microglia were engulfing the transplanted stem cells (human mitochondria^+^/Iba1^+^ cells, as indicated by white arrows). (B) Quantitative data of Iba1-positive cells in different regions. (C) Quantitative analysis of human mitochondria and Iba1 double positive cells. Data are presented as mean ± SEM (*n* = 4–5 animals/group), *P < 0.05 (one-way analysis of variance). DAPI: 4′,6-Diamidino-2-phenylindole; DG: dentate gyrus; Iba1: ionized calcium-binding adapter molecule 1; ICV: intracerebroventricular; MGE-NSC: metabolic glycoengineering modified neural stem cell.

To investigate microglia recruitment, a protective process mediated by integrins and lectins where microglial cells surround vulnerable brain cells (Neumann et al., 2008), we employed immunostaining for Iba1 and human mitochondria in various brain regions post-CA. The Iba1^+^ microglia were minimally detected in the brainstem and most activated in the cortex, as presented by the lowest Iba1 intensity in the brainstem and the greatest Iba1 intensity in the cortex in both treatment groups (**Figure 8A** and **B**). Microglia activation, as evidenced by Iba1 expression, was markedly suppressed in the intranasal group compared with the ICV group in the regions of CA3 (intranasal *versus* ICV: *P* = 0.015; **Figure 8A** and **B**) and DG (intranasal *versus* ICV: *P* = 0.034; **Figure 8A** and **B**).

Furthermore, we compared the number of Iba1^+^/human mitochondria^+^ cells in multiple brain areas between the intranasal and ICV groups. In the ICV group, more colocalized cells were detected in the region of DG than in the intranasal group (intranasal *versus* ICV: *P* = 0.038; **Figure 8A** and **C**), suggesting that more transplanted MGE-NSCs might be engulfed by microglia in the ICV group.

### Angiogenesis after cell transplantation in the brain after cardiac arrest

**Figure 9:**
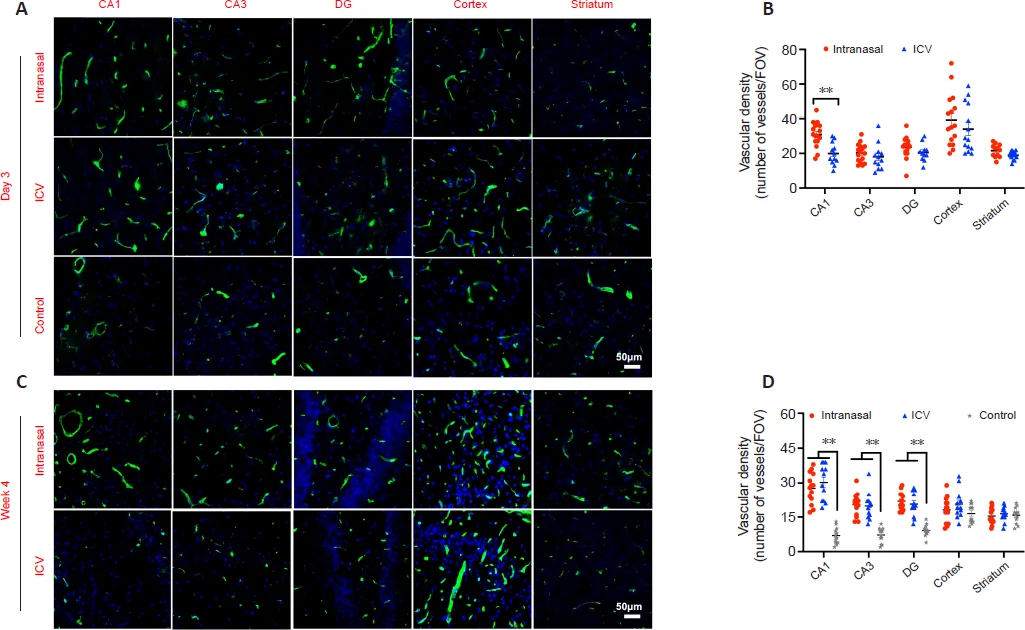
MGE-NSCs enhanced vascular angiogenesis after CA-induced ischemic brain injury. (A) Representative figures of vascular structure (RECA1, green) in different regions 3 days after cardiac arrest/cardiopulmonary resuscitation among the intranasal, ICV, and control groups. Nuclei were counterstained with DAPI (blue). (B) Quantitative results showed that treatment of MGE-NSCs promoted angiogenesis in CA1, CA3, and DG compared with the control group. (C) Representative photographs of vascular structures (RECA1, green) in different regions 4 weeks after cardiac arrest/cardiopulmonary resuscitation. Nuclei were counterstained with DAPI (blue). (D) Quantitative data of vascular density in different regions. Data were presented as mean ± SEM (*n* = 4–5 animals/group), **P < 0.01 (one-way analysis of variance). DAPI: 4′,6-Diamidino-2-phenylindole; DG: dentate gyrus; FOV: field of view; ICV: intracerebroventricular; MGE-NSCs: metabolic glycoengineering modified neural stem cells.

Vascular density was evaluated on days 3 and 28 after ROSC in several different regions in brains with severe and very severe injuries, respectively (**Figure 9A** and **C**). Three days following ROSC, we observed an increased number of RECA1^+^ endothelial cells in treatment groups compared with the CTL group in regions of CA1 (intranasal *versus* CTL: *P* < 0.001; ICV *versus* CTL: *P* < 0.000; **Figure 9A** and **B**), CA3 (intranasal *versus* CTL: *P* < 0.001; ICV *versus* CTL: *P* < 0.000; **Figure 9A** and **B**) and DG (intranasal *versus* CTL: *P* < 0.001; ICV *versus* CTL: *P* < 0.001; **Figure 9A** and **B**), indicating enhanced vascular density after MGE-NSC therapy via both ICV and intranasal admin istration. However, no statistical difference was detected between intranasal and ICV groups (*P* > 0.05; **Figure 9A** and **B**). Four weeks post-ROSC, infusion of NSCs intranasally achieved elevated vascular density in CA1 compared with the method of ICV injection (intranasal *versus* ICV: *P* < 0.001; **Figure 9C** and **D**). These results collectively indicate that intranasal administration is more, or at a minimum equally, effective as ICV administration at promoting angiogenesis.

### Metabolic glycoengineering-modified neural stem cells boost synaptic plasticity in the hippocampus

**Figure 10:**
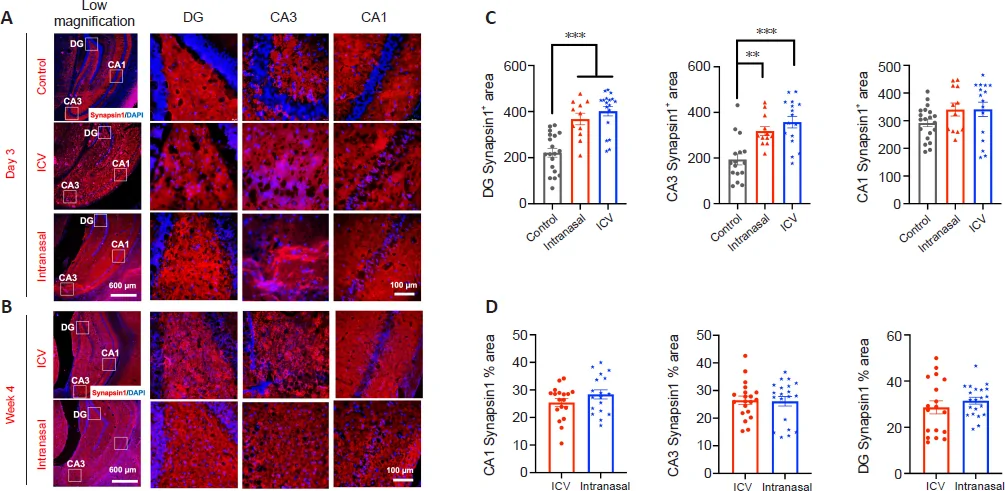
Synaptic plasticity in the hippocampus. (A, B) Representative figures of synapse puncta (synapsin 1, red) in the hippocampus at 3 days (A) and 4 weeks (B) after cardiac arrest/cardiopulmonary resuscitation. The images at low magnification show the anatomical locations of the hippocampal CA1, CA3, and DG. Nuclei were counterstained with DAPI (blue). (C, D) Quantitative data of synapsin 1-positive area in the hippocampus at 3 days (C) and 4 weeks (D). Data are presented as mean ± SEM (*n* = 4–5 animals/group), **P < 0.01, ***P < 0.001 (one-way analysis of variance). DAPI: 4′,6-Diamidino-2-phenylindole; DG: dentate gyrus; ICV: intracerebroventricular.

To investigate how transplanted MGE-NSCs affect synaptic remodeling following CA in models of severe and very severe brain injury, we immunostained synaptic terminals with antibody recognized synapsin 1, a mature presynaptic marker. In the CA3 and DG regions of hippocampus, we observed a notable reduction in fluorescence positive area in control animals on day 3 after CA. The immunohistochemical analysis revealed enhanced synaptic density following treatment with MGE-NSCs, as measured by synapsin 1-positive area. This enhancement was most pronounced within two specific hippocampal zones - the DG and CA3 subfield in the intranasal and ICV treatment groups (**Figure 10A** and **C**). Notably, there were no discernible differences between the Intranasal-NSCs and ICV-NSCs in rats with very severe brain injury at 3 days (**Figure 10A** and **C**) or in those with severe brain injury at 4 weeks (**Figure 10B** and **D**), suggesting that both intranasal and ICV administration have similar effectiveness in promoting synaptic terminal regeneration and plasticity.

To investigate the synaptic integration of transplanted MGE-NSCs in the hippocampus, we performed immunofluorescence staining for synapsin 1, STEM101, and DAPI at 4 weeks post-transplantation. Human-derived cells (STEM101^+^) were observed in the hippocampus of the intranasal and ICV group, with some cells showing clear colocalization with the synaptic marker synapsin 1 (**Additional Figure 4**). These synapsin 1/STEM101 double-positive signals suggest that transplanted MGE-NSCs not only survived but may also have functionally integrated into the host neuronal network via synaptic connections, suggesting that delivery of MGE-NSCs enhances synaptic incorporation and functional connectivity in the ischemia-injured hippocampus.

## Discussion

The current study presents a pioneering investigation into identifying the most effective direct delivery routes for MGE-NSCs to the severely and very severely injured brain following CA, a complex and challenging neurological disorder in clinical practice. Application of MGE-NSCs through different delivery methods to the brain, as exemplified by ICV and intranasal administration in this study, both exerted delivery-specific short-term and long-term beneficial effects on CA-induced global cerebral ischemia. Importantly, this study unveiled the specific fates of MGE-NSCs administered through intranasal or ICV routes. Together, these results establish a scientific foundation for future clinical translation of our novel MGE-based regenerative approach to treating ischemic brain injury post-CA.

This study augments our previous research where we compared an indirect method-intranasal with a direct delivery method-ICV for MGE-NSC therapy after CA. In our earlier studies, we demonstrated that transplanted NSCs exerted protective effects against neuronal ischemia and facilitate neurological recovery of the moderate brain injury following 8-minute CA (Wang et al., 2021, 2023). Next, we adopted an MGE-based approach using thiol-modified monosaccharide analogs to modify the surface of stem cells (Du et al., 2021, 2022); this approach enhances cell adhesion, transcription, and signal responses (Du et al., 2021), thereby promoting the anchoring of therapeutic cells within the injured region. Moreover, our MGE approach rewires cellular metabolism to promote neuronal fates (Dammen-Brower et al., 2025) and stimulates cell differentiation (Du et al., 2022), potentially enhancing subsequent *in vivo* differentiation. This groundbreaking regenerative approach enables cells binding with surrounding adjacent extracellular components, fostering cell viability and neuronal differentiation *in vitro* to enhance therapeutic benefits after *in vivo* transplantation (Du et al., 2024).

Because the advantages of MGE-NSCs help resolve a key limitation in stem cell-based therapies using naïve cells, specifically, poor functioning when introduced into *in vivo* models, we reasoned that MGE-modified NSCs would show efficacy against a more severe manifestation of CA-induced brain injury that occurs in our 9-minute animal model. Accordingly, we compared this standard severe brain injury model with a more severe 11-minute model of experimental ischemia brain injury post-CA in current research. In tandem, we sought to optimize the delivery method for the cells, which is another critical area that currently hampers clinical translation of stem cell therapies. Systemic administration of stem cells targets damaged cerebral regions ineffectively. For example, intravenous administration often leads to the accumulation of stem cells in peripheral organs such as the liver, spleen, kidneys, and lungs (Liu and Jia, 2024a), while intra-arterial delivery can result in the accumulation of cells within cerebral arteries, potentially leading to cerebral infarction (Liu and Jia, 2024a). CA-induced ischemic brain injury serves as a model for global cerebral ischemia, leading to widespread brain damage to the whole brain. Although intraparenchymal injection delivery cells to brain regions (Zhang et al., 2020), ICV infusion, while invasive, directly delivers therapeutics into the cerebral spinal fluid, thereby circulating effective concentrations into the whole brain, showing advantage over the invasive intraparenchymal injection to specific region when considering this animal model. To provide alternative delivery strategies that avoid these pitfalls, this study focuses on two delivery methods (ICV and intranasal) for targeting NSCs to the brain.

In addition to therapeutic efficacy, safety considerations play a central role in advancing stem cell therapies to clinical settings; the current study helped validate the safety of MGE-NSC therapy. Specifically, in this study, there was no excess mortality in the MGE-NSC treated samples in either the severe or very severe models of brain injury after CA; overall, the main cause of death was heart failure or pulmonary edema after CA. Most non-lethal complications reported in severe and very severe CA were seizure and fever, which are common post-CA syndromes (Dalessio, 2020), and symptoms of seizure disappeared when fever was controlled with acetaminophen. No specialized antiepileptic drugs were used in our present study. Tumor formation and immune rejection are additional major concerns of stem cell therapy (Liu and Jia, 2024a). In this study, no tumorigenesis or severe adverse events were detected after MGE-NSC transplantation, irrespective of the severity of brain injury post-CA. It’s worth noting that our NSCs were derived from a human source but they did not elicit noticeable immune rejection when xenotransplanted into immunocompetent rats. We hypothesize that NSC immunoacceptance was facilitated through modulation of pro- and anti-inflammatory metabolites by the MGE process (Dammen-Brower et al., 2025) that reduces the risk of deleterious immune responses, which is an important consideration for future clinical translation (Jin et al., 2016). 

We demonstrated the superior early therapeutic effects of ICV administration over the intranasal route, showcasing the advantages of short-term effects, such as neurological assessment with aggregated NDS at 6 hours, with ICV-NSC therapy. ICV delivery facilitates direct access of stem cells to the CSF. The movement of CSF helps transport the cells across various regions of the central nervous system (Liu and Jia, 2024a). In a previous study, ICV-administered mesenchymal stem cells (MSCs) were more abundant in hippocampus and temporal cortex on day 3 following ROSC than intra-arterial infusion and femoral venous administration (Leong et al., 2016). We observed that delivery via the ICV route enabled immediate brain targeting, leading to greater cell density within specific regions such as the cortex and hippocampus, the regions vulnerable to ischemia in the cortex and hippocampus at the early stage after resuscitation (Leong et al., 2016), which correlates with better NDS in the short term.

Our findings indicated the superior neuroprotection of intranasal-NSCs in the long-term. In the current study, NDS and sub-NDS analyses revealed that transplanting MGE-NSCs intranasally resulted in significantly better advanced motor function than ICV group at the late time point of 1 week post-surgery, with improved neurobehavior outcomes at later time points, at 2 and 3 weeks after CA. At later time point of week 4, human-mitochondria tracked human NSCs were predominantly observed in the regions surrounding the brainstem and cerebellum, and the number of cells in the above regions significantly exceeded that of the ICV-administrated group. The anatomical arrangement of nerve fibers, which merge into bundles through the cribriform plate to reach the olfactory bulb, provides a route for stem cell administration (Liu and Jia, 2024b). Subsequently, these cells can be further transported to the brainstem along the trigeminal nerve or distributed throughout the brain via CSF circulation and migrated to distant regions in the chronic phase (Crowe et al., 2018; Zhang et al., 2021b).

In this study, NSCs were transported to distant regions including brainstem and cerebellum at 4 weeks. In contrast to ICV delivery, intranasally administered cells did not appear to locate in/near the subventricular zone (van Velthoven et al., 2010), but were instead widely distributed to the injured areas in the ipsilateral hemisphere after rodent ischemic stroke or neonatal hypoxia-ischemia (van Velthoven et al., 2010; Chiu et al., 2018; Thomi et al., 2019; Lu et al., 2022). Our study evaluated cell survival, differentiation, and distribution for the first time by intranasal administration of stem cells in model of global cerebral ischemia induced by CA.

Another important aspect of this study involved characterizing the neuronal differentiation of administered MGE-NSCs within the brain. The enhanced behavioral outcomes were potentially be explained by the transformation of transplanted cells into new neurons, helping to repair damaged brain tissue (Wang et al., 2021). In our previous study (Du et al., 2024), engrafted MGE-NSCs were observed penetrating the injured brain tissue and maturing into neurons within these regions. Here, in the current study, our findings indicate a significant increase in the number of MGE-NSCs differentiating into neurons expressing the functional neuronal protein MAP2, contributing to the improved neuronal survival in hippocampal DG and CA3 areas versus control conditions.

Functionally, animals receiving MGE-NSC therapy demonstrated enhanced vascular formation capabilities, evidenced by elevated levels of RECA1 immunoreactivity across hippocampal areas. The treatment group also showed notable improvement in synaptic modification capacity. These functional enhancements suggest a possible compensatory mechanism that could help counteract the typical axonal and synaptic deterioration that typically occurred following CA-induced ischemic brain injury. These functional advantages hold considerable clinical significance, considering the longstanding challenges associated with irreversible brain injury and neurological impairment after CA.

The synapsin protein, synapsin 1, serves as a key marker for synaptic terminals and plays a crucial role in the formation of synapses and the regulation of neurotransmitter release (Colón-Ramos, 2009). In our earlier research (Du et al., 2024), we examined the expression of the general synaptic marker synapsin 1 on hMT-positive transplanted MGE-NSCs in the rat brain. Four weeks after transplantation, synapsin 1-positive puncta were observed surrounding the hMT-positive cells within the MGE-NSC group. This provided evidence of their differentiation into neurons and functionally successful integration into the host brain tissue through synaptic connections. In this study, the localization of transplanted STEM101-positive NSCs and synapsin 1^+^ puncta potentially suggested the functional integration of transplanted MGE-NSCs into host neural circuits through synapse. Additionally, the paracrine effects of the transplanted cells represent another important mechanism contributing to behavioral and histological improvements. Further investigation is necessary to elucidate the specific mechanistic aspects underlying these effects.

Our present research demonstrated elevated numbers of Iba1-expressing microglial cells specifically within the CA3 and DG in subjects that received ICV treatment compared with the intranasal group, indicating that ICV infusion induced higher response of microglia recruitment in the CA brain. ICV is an invasive method of stem cell delivery and can cause localized injury at the injection site (Zhang et al., 2020; Liu and Jia, 2024a). This exogenous injury can further contribute to recruitment and proliferation of microglia in addition to global ischemic injury, which may be the reason for more Iba1-positive microglia in ICV group than in the non-invasive intranasal group post-CA.

From a mechanistic standpoint, microglial activation, characterized by enlarged soma and retracted processes, are involved in phagocytosis under pathological conditions. A preclinical study in rats showed the localization of CD11b-positive macrophages and GFP-positive transplanted MSCs in corpus callosum after ischemic stroke, indicating the engulfment of transplanted MSCs by macrophages (Duan et al., 2017), which is consistent with other studies (Kim et al., 2020; Preda et al., 2021). Immunofluorescence staining has also indicated the phagocytosis of NSCs by Iba1 and CD68 double-positive activated microglia in the brain (Hoshi et al., 2021). Our findings suggest a similar pattern of phagocytosis might occur after MGE-NSC transplantation post-CA, with ICV infusion leading to greater microglia recruitment, which subsequently resulted in more transplanted MGE-NSCs being phagocytized compared with intranasal delivery. The greater phagocytosis observed in the ICV group may contribute to fewer transplanted NSCs remaining in the brain during the late phase of recovery. These results align with the poorer neurological outcomes observed in the ICV group compared with the intranasal group at long-term time points. The detailed biological mechanisms underlying the differing effects observed after transplantation via these two routes will require further investigation in future studies.

The 3-day and 4-week time points post-resuscitation were strategically chosen to evaluate both the early to late functional outcomes and safety of MGE-NSC therapy. This 4-week period (Marsteller et al., 2007) allows us to observe significant integration of transplanted cells and their potential therapeutic effects, such as enhanced neural network stabilization and behavioral improvements, in a temporally relevant window post-injury (Du et al., 2024). While the 4-week time point provides critical insights into the safety and therapeutic effects of MGE-NSC therapy, we recognize that a longer-term follow-up may provide additional valuable data regarding sustained efficacy, late-emerging effects, and additional safety considerations. Future studies are needed with extended observation periods at later time points to better understand the long-term integration and performance of MGE-NSCs, further informing future clinical translation.

Our study utilizes a rat model of asphyxia CA, which effectively replicates several critical aspects of human brain injury following CA, such as cerebral ischemia, neuronal loss, and cognitive deficits. However, interspecies differences persist, limiting direct extrapolation to human conditions. Future research is needed to incorporate other models including larger animal models to offer closer physiological and anatomical similarities to humans. These studies aim to validate and extend our findings, thereby bridging the gap towards clinical application.

Our MGE-NSC therapy at 3 hours post CA/CPR enhanced neurological recovery. The optimal delivery time in NSC treatment for ischemia is still controversial with a range of 2 hours to 7 days (Zhang et al., 2020; Liu and Jia, 2024a). Our results suggested that novel MGE’d stem cell therapy in the very early phase (3 hours) post-ROSC is feasible and effective, with superior neurological cognitive functions and fewer behavioral markers associated with mood disorders in rats. A single dose of intranasal administration exhibited long-lasting beneficial effects, even 4 weeks post CA/CPR. However, a single dose in the ICV route was not as therapeutically effective as intranasal infusion, although ICV delivery of MGE-NSCs also increased NDS score. Fewer NSCs survived in the brain 4 weeks after ROSC when administered via ICV, which indicates higher concentration or repeated dosage may be needed to gain a similar therapeutic effect. While repeated administration is out of the scope of current study, the invasive nature of ICV administration reduces the feasibility of repeated doses, further supporting intranasal administration as we move forward to clinical translation. Determining the optimal therapeutic window and dosages for stem cell therapy remains a key area of interest, pending further investigation.

## Conclusions

The pathophysiology of CA-induced ischemic brain injury is an intricate progressive pathology, often leading to enduring cognitive impairments for which effective treatments remain elusive; this study unveils a novel and promising MGE-NSC-based therapeutic approach to overcome these limitations. Furthermore, multiple benefits and pitfalls of ICV versus intranasal administration in mitigating CA-induced neuropathology are outlined in the context of vascular and synaptic deficits, indicative of short-term and long-term effects following CA, respectively. Together, this information constitutes a hopeful therapeutic avenue for either preventing or treating CA-induced neurodegenerative conditions, underscoring the prospect of treatment strategies tailored to different phases of post-resuscitation care.

## Additional files:

***Additional Figure 1:*** *The electrophysiological characteristics.*

Additional Figure 1 The electrophysiological characteristics.
(A) EEG-IQ analysis through the first 6 hours after severe brain injury between the intranasal and ICV group. (B) Overall trend of EEG recovery. (C) Representative EEG waveforms before surgery, during cardiac arrest time and after resuscitation. Data are presented as mean ± SEM (*n* = 8 animals/group) (one-way analysis of variance). CA: Cardiac arrest; ECG: electrocardiogram; EEG: electroencephalogram; ICV: intracerebroventricular; IQ: information quantity; ROSC: return of spontaneous circulation

***Additional Figure 2:*** *Assessment of sensory and brainstem function after metabolic glycoengineering-modified neural stem cell transplantation in cardiac arrest rats with severe brain injury (4-week long-term study).*

Additional Figure 2 Assessment of sensory and brainstem function after metabolic glycoengineering-modified neural stem cell transplantation in cardiac arrest rats with severe brain injury (4-week long-term study).
Sub-NDS/ sensory function (A) and brainstem outcome (B) comparison between the intranasal and ICV groups at five time points post-return of spontaneous circulation. No significant difference was observed in terms of sub-NDS (*P* > 0.05). Data are depicted as median, 25^th^-75^th^ percentile (*n*=8 rats per group). ICV: Intracerebroventricular; NDS: neurological deficit score.

***Additional Figure 3:*** *Neurological functions were ameliorated with the treatment of neural stem cells in cardiac arrest rats with very severe brain injury (3-day short-term study).*

Additional Figure 3 Neurological functions were ameliorated with the treatment of neural stem cells in cardiac arrest rats with very severe brain injury (3-day short-term study).
Neurological function was assessed using NDS (A) and survival NDS (B) among the control, intranasal and ICV groups. There was no significant difference among the three groups in average survival time (C) and 72-hour cumulative survival rate (D). Data in A and B are presented as median, 25^th^-75^th^ percentile (*n* =7 animals/group). *P < 0.05 (nonparametric test of Kruskal-Wallis one-way analysis of variance). Data in C are shown as mean ± SEM (*n* = 7 animals/group), using one-way analysis of variance. Data in D are presented as cumulative survival rate (*n* = 7 animals/group), using Kaplan-Meier method. CTL: Control; ICV: intracerebroventricular; NDS: neurological deficit score.

***Additional Figure 4:*** *Immunofluorescence analysis of human NSCs-derived synaptic integration in the hippocampus 4 weeks after transplantation.*

Additional Figure 4 Immunofluorescence analysis of human NSC-derived synaptic integration in the hippocampus 4 weeks after transplantation.
Representative images show co-labeling of synapsin 1 (red), human-specific STEM101 (green), and nuclear DAPI (blue) in the hippocampus from rats receiving MGE-NSCs via intranasal (left) or ICV (right) routes. The colocalization of synapsin 1 and STEM101 (indicated by white arrows) suggests potential synaptic integration of transplanted human NSCs with host neural circuits. DAPI: 4′,6-Diamidino-2-phenylindole; ICV: intracerebroventricular; MGE-NSC: metabolic glycoengineering modified NSC; NSC: neural stem cell.

***Additional Table 1:*** *Neurological deficit scores in the intranasal and ICV groups.*

***Additional Table 2:*** *Sub-neurological deficit scores (motor) in the intranasal and ICV group.*

***Additional Table 3:*** *Sub-neurological deficit scores (sensory) in the intranasal and ICV groups*

***Additional Table 4:*** *Sub-neurological deficit scores (brainstem function) in the intranasal and ICV groups.*

***Additional Table 5:*** *Body weight (g) comparison in the intranasal and ICV groups.*

**Additional Table 5:** Body weight (g) comparison in the intranasal and ICV groups

	Intranasal	ICV	*P* value
Baseline	358.50 + 6.48	355.57 + 13.18	0.732
24 h	323.12 + 5.34	322.57 + 7.67	0.953
48 h	301.50 + 7.48	313.16 + 8.19	0.318
72 h	293.62 + 9.34	305.00 + 11.09	0.456

Data are expressed as mean ± SEM. ICV: Intracerebroventricular.

## Data Availability

*All data relevant to the study are included in the article or uploaded as Additional files.*
